# Shwachman-diamond syndrome: A case report

**DOI:** 10.1097/MD.0000000000024712

**Published:** 2021-02-19

**Authors:** Huihan Tan, Dequan Su, Zhiqiang Zhuo

**Affiliations:** a Xiamen Children's Hospital; b Infectious Department, Xiamen Children's Hospital, Xiamen, Fujian, China.

**Keywords:** heterozygous mutations, SBDS genes, shwachman-diamond syndrome

## Abstract

**Rationale:**

The aim of this study was to analyze the genetic abnormalities and clinical manifestations of Shwachman-Diamond syndrome (SDS).

**Patient concerns:**

A Chinese infant with elevated transaminase and a novel mutation at of sbdsc.258 +2T>C and c.184a>Tc.292G>A.

**Diagnoses:**

The female patient was 5 months’ old at onset, with elevated transaminase as the first manifestation accompanied by restricted growth and development and oily stool. After sequencing the blood samples from patients and their parents, the heterozygous mutations of sbdsc.258 +2T>C and c.184a>T were detected.

**Interventions:**

After admission, the patient was provided compound glycyrrhizin, Newtide formula milk supplemented with probiotics, fat-soluble vitamins, oral medication to adjust the spleen and stomach, and other symptomatic treatments.

**Outcomes:**

The stool traits improved, and the levels of liver function transaminases decreased compared with before.

**Lessons:**

SDS is a rare disease with a variety of clinical manifestations. Pancreatic exocrine dysfunction, blood system manifestations, and bone abnormalities are common clinical manifestations, and genetic testing is helpful for diagnosis.

## 1 Introduction

Shwachman-Diamond syndrome (SDS) is an autosomal recessive genetic disease first described by Nezelof and Watchi in 1961.^[1]^ SDS is rare and only several hundred cases have ever been reported. The clinical phenotype is mainly pancreatic exocrine dysfunction, an abnormal blood system, and skeletal abnormalities. There are also reports of cardiac abnormalities and immune dysfunction.^[2]^ More than 90% of cases are caused by mutations in the *Shwachman-Bodian-Diamond syndrome* (*SBDS*) gene on chromosome 7q11.^[3]^ In this study, the clinical and genetic analysis of a child with SDS and a review of relevant domestic and global literature were conducted to explore the clinical manifestations and genetic characteristics of this disease.

## 2 Case report

### 2.1 General information

A 5-month 24-day-old baby girl came to our hospital because of “9 days of abnormal liver function.” She was first admitted to another hospital for “bronchial pneumonia.” During her hospitalization, she was found to have increased transaminases (alanine aminotransferase [ALT] 122–157 U/L, aspartate aminotransferase [AST] 75–120 U/L) and was treated with ceftazidime injection, compound glycyrrhizin injection, and fluid replacement. Her liver function did not improve. She occasionally coughed, easily cried, and had a poor appetite. She produced stools 3 to 5 times a day that were mushy and oily. She was the third child of healthy and unrelated parents and was born on the 37^th^ week of gestation with a birth weight of 2900 g. She had 2 healthy elder sisters. At the time of admission, the patient was 60 cm tall and weighed 5000 g; thus, she was below the third percentile. Her lung examination was basically normal. She had no hepatomegaly, splenomegaly, or enlarged lymph nodes. This study was approved by the ethics committee of the Xiamen Children's Hospital, and written consent for its publication was provided by the patient's parents.

### 2.2 Laboratory examination

Her white blood cell count was 8.68 × 10^9^ cells/L, absolute neutrophil count was 3.12 × 10^9^ cells/L, number of platelets was 560 × 10^9^ cells/L, and hemoglobin concentration was 114 g/L. Her 25-hydroxyvitamin D levels were low (29.7 ng/mL). AST (114 U/L) and ALT (163 U/L) levels were elevated, and total cholesterol was 2.16 mmol/L. Prothrombin time; albumin levels; creatine kinase; serum IgA, IgG, IgM; and other serum biochemical parameters were within normal ranges. The patient was positive for fecal fat globules.

### 2.3 Imaging data

Digestive system ultrasound found no obvious abnormalities in the gallbladder, pancreas, or spleen, and no obvious abnormal echoes were seen in the adrenal glands.

### 2.4 Genetic test results

**Figure 1:**
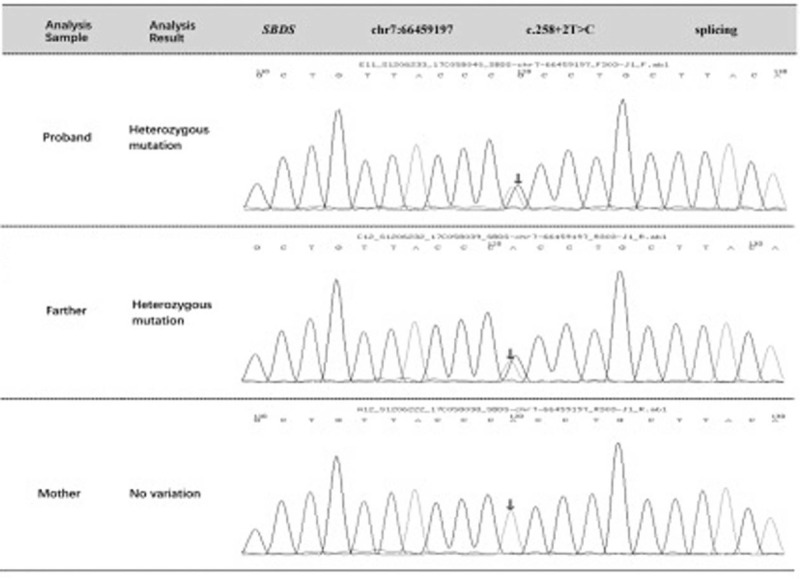
Sanger sequencing results for the children and their parents (the arrows indicate the positions of mutations between the tested and normal.

**Figure 2:**
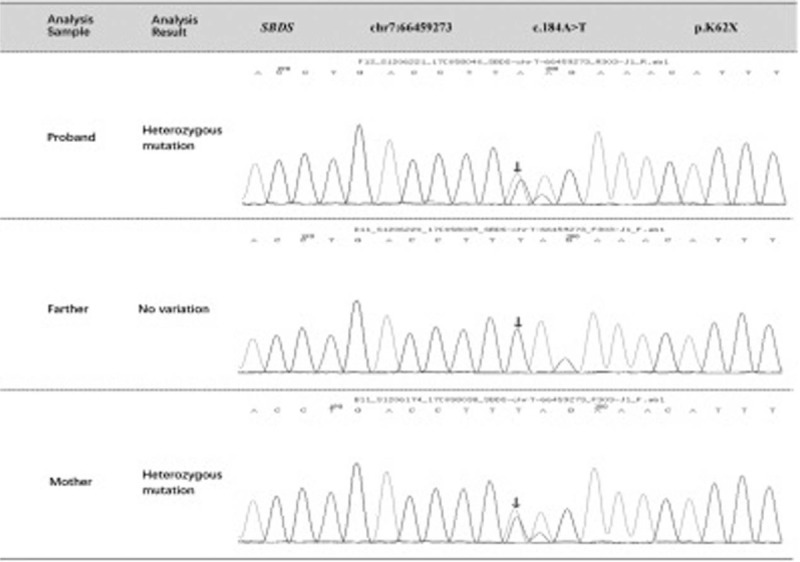
Sanger sequencing results for the children and their parents (the arrows indicate the positions of mutations between the tested and normal.

The patient's blood sample was analyzed, and the results showed 2 heterozygous mutations in the *SBDS* gene (Table 1). First, c.184A>T heterozygous mutations, resulting in amino acid changes producing p.K62X mean nonsense mutations. The transformation of A to G of 185 non-pathological silencing nucleotides in SBDS gene was also detected in the heterozygosity (Fig. 1). On family analysis and verification, there were no mutations at this locus in the patient's father, and heterozygous variation was found at the locus in the mother. The frequency of this mutation in the normal population database was 0.00190. Second, c.258+2T>C synergistic mutations are splicing mutations, resulting in the splicing of amino acids. After family analysis and verification, the parent of the examinee had heterozygous variation and the mother had no variation at the locus. The frequency in the normal population database was 0.00930 (Fig. 2).

**Table 1:** SBDS gene analysis of children.

Gene	chromosomal location	Mutation information	Homozygous/Hybrid	Father	Mother	Normal population frequency	AMG pathogenicity analysis	Disease/phenotype	Inheritance
SBDS	chr7:66459273	c.184A>T (p.K62X)	Hybrid	No variation	Hybrid	0.0019	Pathogenic	1. Shwachman-Diamond syndrome 2. Aplastic anemia, susceptibility	1.AR2.-
SBDS	chr7:66459197	c.258+2T>C(splicing)	Hybrid	Hybrid	No variation	0.0093	Pathogenic	1. Shwachman-Diamond syndrome 2. Aplastic anemia, susceptibility	1.AR2.-

### 2.5 Treatment

After admission, the patient was provided compound glycyrrhizin, Newtide formula milk supplemented with probiotics, fat-soluble vitamins, oral medication to adjust the spleen and stomach, and other symptomatic treatments. The stool traits improved, and the levels of liver function transaminases decreased compared with before.

### 2.6 Literature review

We performed a search for SBDS-related literature in PubMed published from January 2014 to December 2018, and, after screening and exclusion of duplicate articles, 12 of them met the standards, and 119 cases were included.^[4–16]^ Clinical manifestations (Table 2) and genetic test results (Table 3) were analyzed.

**Table 2:** Primary manifestations of 119 patients.

Manifestations	No. patients	Rate
Pancreas malfunction	26	21.85%
Failure to thrive	18	15.13%
Elevation of liver enzymes	17	14.29%
Diarrhea	10	8.40%
Vomiting	2	1.68%
Abdominal pain	1	0.84%
Constipation	1	0.84%
Skeletal Anomalies		
Osteoporosis	20	16.81%
Abnormal frame	1	0.84%
Metaphyseal dysplasia cartilage	1	0.84%
Blood system performance		
Pancytopenia	17	14.29%
Neutropenia	10	8.40%
Neutropenia, Thrombocytopenia	3	2.52%
Thrombocytopenia	1	0.84%
Anemia	1	0.84%
AML	1	0.84%
MDS	3	2.52%
Hemophilia A	1	0.84%
Other complications		
Eczema	2	1.68%
Liver cirrhosis	1	0.84%
Psoriasis	1	0.84%
Temporary visual impairment	1	0.84%
Hypothyroidism	1	0.84%
Others		
Loss of consciousness	1	0.84%
Hypotonia	1	0.84%

**Table 3:** Molecular detection of SBDS gene mutations in 119 patients.

Molecular testing location	No. of patients	Rate
c.183–184TA>CT/c.258+2T>C	99	83.19%
c.258+2T>C/c.460–1G>A	4	3.36%
c.258+2T>C/c.292–295delAAAG	2	1.68%
c.183–184TA>CT/c.258+2T>C /c.201A>G	2	1.68%
c.258+2T>C/c.97A>G	2	1.68%
c.258+2Tc	2	1.68%
c.183–184TA>CT/c.258+2T>C/c.201A>G /c.141C>T	1	0.84%
c.258+2T>C/c.259–1G>A	1	0.84%
c.258+2T>C/c.428C>G	1	0.84%
c.183–184TA/c.79T>C	1	0.84%
c.258+2T>C/unknown	1	0.84%
Not detected	3	2.52%

## 3 Discussion

SDS is a rare, autosomal recessive genetic disease with an incidence of 1:76,000,^[17]^ and 90% of patients have mutations in the SBDS gene on chromosome 7q11. Other patients with a clinical diagnosis of SDS may not have known mutations. The *SBDS* gene encodes a protein that participates in microtubule stabilization and actin polymerization, and it plays roles in bone marrow cell proliferation, mitosis, and the matrix microenvironment.^[18]^ There is increasing evidence that SBDS also has ribosomal function: SBDS may interact with certain ribosomal subunits and play an important role in ribosome maturation.^[19,20]^ Most SBDS gene mutations cause the SBDS protein to be truncated.^[21]^ SBDS-deficient mouse models showed ribosomal synthesis defects and protein translation insufficiency, which further indicated that SDS is a ribosomal disease^[22]^; however, SBDS deficiency did not change whole-cell telomerase activity, suggesting that SBDS may not be involved in telomerase biogenesis. Liu et al's research showed that SBDS maintains the human telomere system by regulating telomerase recruitment and may help to determine the appropriate research approach for developing therapeutic targets for SDS and other telomerase-related diseases.^[23]^

According to the literature review, the most common genetic mutations in patients with SDS are c.258+2T> C and c.183–184TA> CT. There were no SBDS-gene mutations detected in about 2.52% of children with the condition. The patient described in this report had heterozygous mutations, c.258+2T>C and c.184A>T. According to the ACMG guidelines, the mutations were initially determined to be the pathogenic variant PVS1 + PS1 and the pathogenic variant PVS1 + PS1 + PM1 + PM2. So far, no correlation between SDS genotype and phenotype has been observed.^[24,25]^

SDS usually occurs in infants and young children. Its performance is complex and diverse, involving the pancreas, liver, kidneys, teeth, bones, blood system, and immune system, and some children also present with skin disorders. Over time, the clinical manifestations among patients, and even within the same person, vary greatly.^[18]^^.^Clinically, SDS has 3 main manifestations: pancreatic exocrine dysfunction, blood system performance, and skeletal abnormalities.

Exocrine pancreatic failure in children is usually due to cystic fibrosis, but SDS is the second most common cause. Therefore, pancreatic dysfunction is considered an important part of the diagnosis of SDS. SDS pancreatic exocrine insufficiency often occurs within 1 year after birth^[25]^ and is caused by pancreatic acinar cell depletion and fat infiltration. The main manifestations are decreased pancreatic elastase content, reduced fat-soluble vitamin content, and increased fecal fat content, all of which can all be used to diagnose pancreatic failure. In terms of imaging, MRI may show pancreatic lipomas.^[26,27]^ Furthermore, 60% of patients with SDS have elevated liver transaminase. About 15% of people show hepatomegaly, which may be caused by steatosis (“fatty liver”).^[2]^

In the present case, the main manifestation was abnormal transaminase elevation. After the patient was born, her feces were oily and routinely showed fat globules without accompanying liver enlargement. After adjustment of feeding and hepatoprotective treatment, the stool characteristics gradually improved and transaminase decreased. Studies have shown that elevated transaminase and liver enlargement gradually improve at around 5 years of age, which may cause difficulties for differential diagnosis.^[25,28]^ In untreated pancreatic insufficiency, fat-soluble vitamin levels and the vitamin K-dependent prothrombin time may be abnormal and, therefore, should be monitored. Hematological abnormalities are also common in patients with SDS, including cytopenia, varying degrees of myelodysplastic abnormalities, and an increased risk of acute myeloid leukemia. The most common single-cell reduction is leukopenia, which causes repeated infections by viruses and bacteria, leading to problems such as sinusitis, pneumonia, osteomyelitis, and septicemia. Some patients with SDS may also have repeated infections without obvious neutropenia, which is related to the impairment of neutrophil chemotaxis in SDS patients.^[2]^ Patients may develop bleeding, due to thrombocytopenia, that is sometimes life-threatening. Large-cell or normal-cell anemia can also be seen in up to 80% of patients.^[11]^ The French Severe Chronic Neutropenia Registry found that 41 of 102 patients with SDS (40%) showed significant blood symptoms, of which 21 had persistent severe cytopenias (9 were malignant and 9 nonmalignant, and 3 cases progressed from nonmalignant to malignant). The study suggested that the risk of serious and potentially fatal complications in SDS is very high, as high as 25% by the age of 20. Hematocytopenia, anemia (including mild forms), or thrombocytopenia occur within 3 months after birth, and they are considered high-risk factors for serious hematological complications (malignant or nonmalignant).^[29]^ Almost every patient with SDS has skeletal abnormalities, including short stature, thoracic abnormalities, spinal and finger deformities, and spondylolisthesis. Severe osteoporosis and osteomalacia usually occur, most likely secondary to the impaired absorption of vitamin D and vitamin K, resulting in increased spinal and peripheral fractures.^[24]^ Many children with SDS suffer from neurological, learning, and/or behavioral difficulties.^[5]^ Studies have found that patients with SDS may have significant barriers to advanced language skills, intellectual reasoning, academic performance, and perception, including reasoning and visual motor skills.^[30]^

The current treatment for SDS is mainly oral pancreatin and fat-soluble vitamins to supplement pancreatic exocrine pancreatic insufficiencies. Erythrocyte transfusion, platelet, and granulocyte colony-stimulating factors may be considered for anemia, neutrophilia, and thrombocytopenia. For severe pancytopenia, those with MDS or acute myeloid leukemia should be considered for hematopoietic stem-cell transplantation. Meanwhile, attention should be given to testing the blood routine, growth and development, nutritional status, bone marrow and bones and joints, and neuropsychological issues, among others.

After admission, the patient was fed compound glycyrrhizin and Newtide formula milk supplemented with probiotics, fat-soluble vitamins, and children's Fupi granules to regulate the spleen and stomach. The patient's vital signs were stable, her body temperature was normal, and there was no vomiting. Liver function was reviewed, and because the transaminase was lower than before, the patient was discharged.

## 4 Conclusions

At present, there is no specific treatment for SDS and mainly symptomatic treatment is provided. Therefore, in future, we require further research into the disease to clarify the cause and find effective treatment methods. The incidence of SDS is low, the symptoms of some patients are atypical, and clinical understanding is insufficient. Over time, clinical performances among patients and even within the same person vary greatly, which is a challenge for diagnosis and treatment. For children with recurrent liver dysfunction of an unknown etiology and limited growth, it is recommended that genetic testing is performed as early as possible to avoid a delay in treatment and to promptly detect the risk of MDS or acute myeloid leukemia development.

## Acknowledgments

The authors thank the patient and her family for allowing us to use the medical documentation and information that led to the present article.

## Author contributions

**Conceptualization:** Hui-han Tan, Zhi-qiang Zhuo.

**Data curation:** Hui-han Tan, De-quan Su

**Formal analysis:** Hui-han Tan

**Methodology:** Dequan Su, Zhi-qiang Zhuo.

**Resources:** Zhi-qiang Zhuo.

**Supervision:** Hui-han Tan, Zhi-qiang Zhuo.

**Validation:** Hui-han Tan

**Visualization:** Dequan Su.

**Writing – original draft:** Hui-han Tan

**Writing – review & editing:** Huihan Tan, Zhi-qiang Zhuo.
